# Microbiome varies by body site in the yellow mud turtle, *Kinosternon flavescens,* from the Chihuahuan Desert

**DOI:** 10.1128/spectrum.03664-25

**Published:** 2026-07-20

**Authors:** Joseph D. Madison, Drew R. Davis, Benjamin W. Genter, Travis J. LaDuc, Carly R. Muletz-Wolz

**Affiliations:** 1 Smithsonian’s National Zoo and Conservation Biology Institute 543127, Washington, DC, USA; 2 Department of Biology, University of Massachusetts Boston 14708, Boston, Massachusetts, USA; 3 Department of Biology, Xavier University of Louisiana 5785, New Orleans, Louisiana, USA; 4 Department of Biology, Eastern New Mexico University 6666, Portales, New Mexico, USA; 5 Biodiversity Collections, Department of Integrative Biology, The University of Texas at Austin 12330, Austin, Texas, USA; 6 Division of Herpetology, Florida Museum of Natural History, University of Florida 3463, Gainesville, Florida, USA; The University of Tennessee Southern, Pulaski, Tennessee, USA; Beijing University of Agriculture, Beijing, China; Henan University College of Life Science, Kaifeng, Henan province, China

**Keywords:** microbiome, turtle, cyanobacteria, *Paludibacter*, desert

## Abstract

Microbiomes are important to the ecology and evolution of their associated animal hosts. These important functions range broadly from individual animal health to population-level adaptations. Turtle-associated microbiomes are under increasing investigation, given turtles’ conservation status and the unique natural history of the shell as an evolutionary-developmental novelty. Many components of the turtle-microbiome interaction remain understudied, including how microbial communities assemble based on host and environmental factors. Here, we hypothesized that age, habitats, and body sites would exhibit significant differential effects on bacterial microbiomes in the Yellow Mud Turtle (*Kinosternon flavescens*) in the Chihuahuan Desert of West Texas, USA. We also hypothesized there would be differential abundance of specific bacterial taxa associated with a recently described, algae-mediated shell disease. Using 16S rRNA amplicon sequencing of 64 turtle samples, we found bacterial community differences among body sites (cloaca, carapace, plastron, and skin), but weaker trends associated with habitat types (ephemeral or permanent-water ponds), and over age gradients (age 4–12+ years). Specifically, the carapace and plastron hosted high bacterial richness compared to the skin and cloaca, and all body sites differed in their bacterial composition across habitat sites. In many individuals, including those with advanced stages of the algae-mediated shell disease (Stage 3 or 4; 25% of sampled turtles), we detected significant differential abundance of specific bacterial taxa, including Cyanobacteria strains. These results are important for informing sampling regimes in long-term studies related to *K. flavescens* ecology and evolution, and for continuing conservation and natural history work associated with turtles broadly.

**IMPORTANCE:**

This research addresses the role of host and environmental factors in shaping bacterial communities in turtles. Specifically, this study is the first to test the hypothesis that body and habitat sites drive microbial community structure and enrichment patterns in the Yellow Mud Turtle, *Kinosternon flavescens*. Turtle body site is shown to be a significant contributor to turtle bacterial community structure and enrichment. This includes the presence of specific taxa that may have a role in a specific algae-mediated shell disease, which is of interest to conservation efforts. Taxa associated with the cloaca across individuals are also identified, as these may have important evolutionary implications. This work will be important to generating hypotheses and guiding methods in future turtle microbiome studies and will also serve as important background information for turtle conservation efforts.

## INTRODUCTION

Host-associated microbial communities, or microbiomes, have an important causal role in their associated animal's ecology and evolution. The complex interactions among microbiomes, animals, and environment over time have implications for our understanding of ecology and evolution broadly. Additionally, understanding these interactions has applied use in dimensions such as animal health and conservation. Of the various animal-microbiome systems of study, there exist vast chasms in baseline information that are useful to any given animal system and their associated habitats. Humans and rodents are overrepresented in these investigations, and for most animal systems, we know nothing about their associated microbiomes (1). One animal system of importance for both its application to basic eco-evolutionary theory and applied dimension of conservation is turtles (order Testudines). The development and evolutionary origins of the turtle shell have been of particular interest to biologists for at least two centuries (2–5), with the evolutionary developmental genetic underpinnings of this novelty having the most recent focus (6, 7). The role of the microbiome in shaping gene expression patterns related to evolutionary novelties and innovations remains an under-explored frontier. Likewise, there remains a significant dearth of work examining the interactions between turtle-associated microbiomes and individual and population health outcomes. This has important implications for ongoing turtle conservation efforts, as numerous species are threatened or facing extinction (8).

Future efforts to understand the role of turtle-associated microbiomes across species and habitats will require large data sets encompassing multiple species across spatiotemporal gradients. To this end, some degree of protocol standardization will be necessary for collecting samples from individual turtles. In designing future studies that will encompass large field sampling efforts, two questions that are of great importance include whether the microbiome is significantly different across body sites and if there are differences among habitats for a given species. While these questions have been previously examined to some extent (9–12), to our knowledge, both body site and habitat differences have not been simultaneously examined in the same turtle species. However, we note that general reptile microbiome research was not comprehensively reviewed here. There are also outstanding questions related to bacterial microbiome changes in turtles in both the course of their lives (age) and with respect to various disease conditions, an important health issue in taxa with implications to conservation efforts (13–15). Over a 20-year monitoring project, an algae (*Arnoldiella chelonum*)-mediated shell disease (hereafter, pitting) noted by Christiansen et al. (14) revealed 100% prevalence in the study population by age 13. Although this condition may not be lethal, this pitting condition subjects the turtles to reduced protection from predators and higher risk of desiccation when moving between ephemeral ponds (D. R. Davis and T. J. LaDuc, unpublished data).

To better aid in our understanding of these questions, we conducted a study comparing microbiomes by body site and habitat type in the Yellow Mud Turtle, *Kinosternon flavescens*, in the Chihuahuan Desert of the southwestern USA. In our models, we also examined age and pitting stage as additional factors of biological interest. We hypothesized based on limited previous work from other turtle species that there would be significant differences between the four body sites examined (carapace, plastron, skin, and cloaca) and among sites (ephemeral or permanent-water ponds). We also hypothesized significant microbial community differences among age and pitting stage. Findings from this study are important both for understanding the role of the microbiome in *K. flavescens* on health and conservation*,* and in protocol design and implementation for future large-scale turtle-microbiome studies.

## MATERIALS AND METHODS

### Study area and species

*Kinosternon flavescens* (Kinosternidae) is a small omnivorous turtle found throughout a large part of the Great Plains of North America, from Nebraska, USA, south through northern Mexico (16). The natural history of *K. flavescens* differs markedly from those turtle species whose microbiomes have been previously studied. This terrestrial species lives in a predominantly arid habitat, only entering bodies of water for a few weeks or months each year during the annual summer monsoon rains. Historically, across this region, these turtles utilized ephemeral pools that were formed in arroyos or shallow depressions across the landscape. In addition to these temporary pools, a few larger, man-made catchment ponds can now be found through the landscape, with some ponds permanently fed with groundwater pumped from wells.

**Fig 1:**
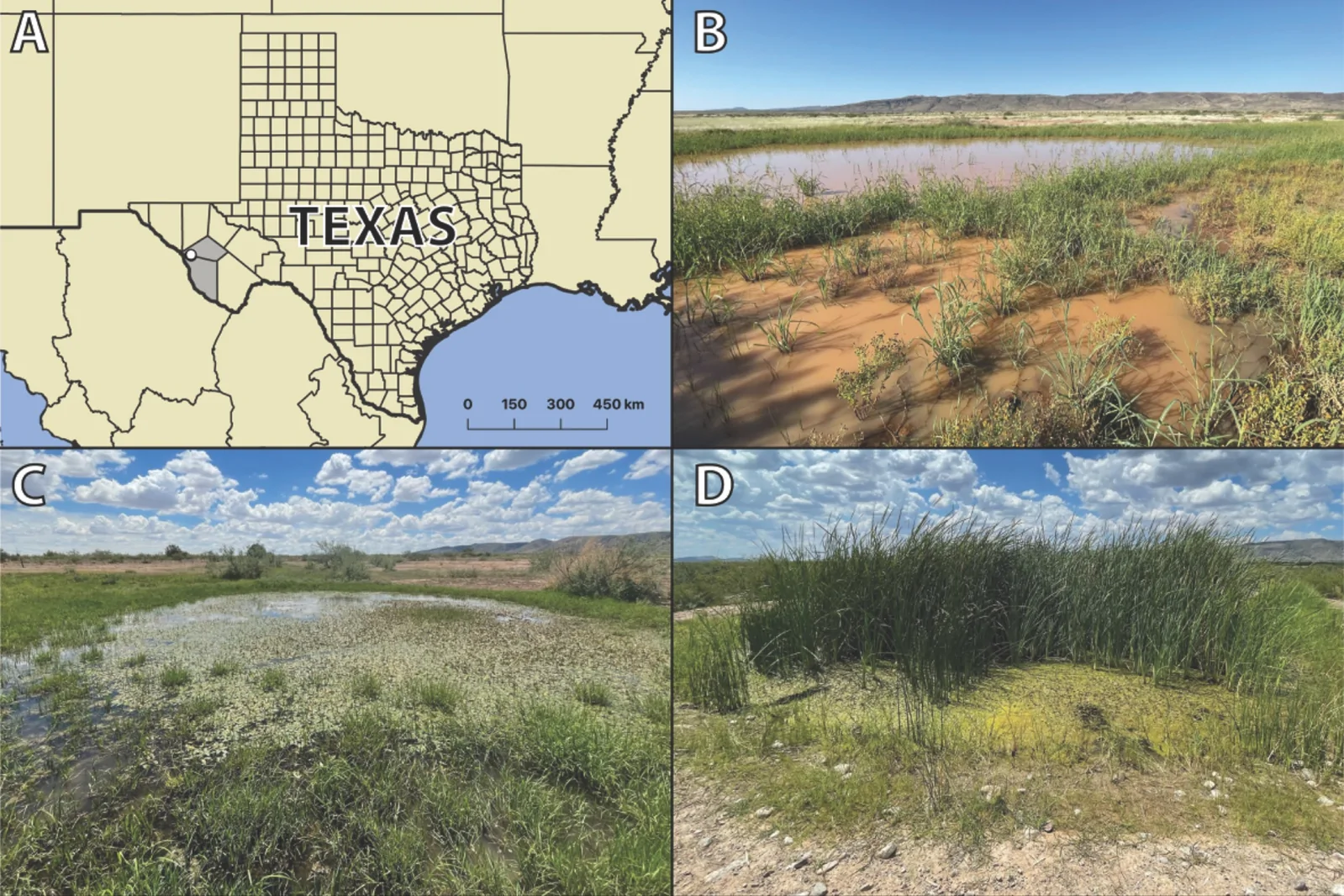
Sampling locations of the three sites for this study: (**A**) map of Texas, USA, indicating the general sampling region in Jeff Davis and Presidio counties (grey highlighting) and sites (white circle). The three sampled sites include: (**B**) Tin House, an ephemeral, constructed pond that serves as a water catchment (*n* = 4 turtles), (**C**) Glidewell, an established, permanent, well-fed, dugout pond (*n* = 8 turtles), and (**D**) Cattail Pond a recently constructed, permanent, well-fed, dugout pond (*n* = 4 turtles). The map was made using QGIS. The software and all layers are open source.

We sampled *K. flavescens* at three different sites on C. E. Miller Ranch near Valentine, Texas, USA. C. E. Miller Ranch is a ca. 13,300 ha private cattle ranch situated in Presidio and Jeff Davis counties and has a long history of biological and herpetological research (17, 18). Recently, research has focused on large populations of *K. flavescens* on the ranch (14). This ranch is situated within the broader Chihuahuan Desert ecoregion, and average annual rainfall at this site is ca. 34 cm, the majority of which falls from June through August. The three sites we sampled included: (i) an ephemeral, constructed pond that serves as a water catchment that collects water during rains as it runs off the Sierra Vieja and into the Valentine Plain (Tin House), (ii) an established, permanent, well-fed dugout pond that serves as a cattle tank (Glidewell), and (iii) a recently constructed, permanent, well-fed dugout pond (Cattail Pond) (Fig. 1).

### Sample collection

Turtle capture and sampling at our localities were conducted over a 5-day period during August 2022. Using partially submerged Promar traps baited with sardines, we captured 72 *K. flavescens,* of which a subset (*n* = 16; File S1) was used for this study. A subset of the 72 was used due to cost and logistical constraints, with four individuals selected from each site representing diverse sex, age, and shell pitting states (*n* = 12), and an additional four individuals selected from Glidewell to better represent the total age distribution at that site. Turtle sample distribution across body site, habitat site, age group, and shell pitting status is summarized in Table S1. Traps were left submerged overnight, and captured individuals were swabbed, processed, and released. First, we swabbed the turtle with a different protocol depending on the body location. For cloacal swabs, we followed previously used methods (19). Specifically, we inserted a sterile rayon-tipped swab (No. MWE113, Medical Wire & Equipment, Corsham, Wiltshire, UK) into the cloaca, rotated fully three times, and gently removed. For plastron and carapace swabs, we followed methods reported by Parks et al. (20). Specifically, we swabbed four defined sites on the plastron (with one swab) and four defined sites on the carapace (with one swab), with each swab site having the swab rotated fully three times. This resulted in 12 (3 rotations across 4 sites) rotations per swab, with separate swabs for carapace and plastron. For skin swabs, we swabbed each of the four limb insertion points (i.e., armpits) with the same swab, with three full rotations of the swab per limb insertion point. This resulted in 12 (3 rotations in each limb insertion point) rotations for one skin swab per turtle. Environmental control swabs (*n* = 3; one per site) were briefly submerged into the pond water and bottom substrate. Air (negative) controls (*n* = 3; one per site) were opened at the corresponding habitat site and briefly exposed before storage. Swab samples were stored individually in sterile microcentrifuge tubes containing 500 µL of 95% ethanol, held on ice in the dark while in the field until transported to a −20°C freezer (<4 h), and then transported to a −160°C liquid nitrogen freezer where they stayed until DNA extraction and purification.

After swabbing, turtles were measured, sexed, given a unique identifying notch on their marginal scutes for future studies (following a modified version of Congdon and Gibbons [21]), and photographed. We also evaluated an algae-mediated shell disease by scoring (Stages 0–4; 0 representing no pitting and 4 representing severe pitting) the presence of pitting on the carapace following a study by Christiansen et al. (14). Age was determined by counting growth rings on the pectoral (plastral) scute (22). Turtles older than 12 years had unreliable age estimates due to the difficulty in counting growth rings and were considered “old” during subsequent analyses. After swab and data collection, we released all individuals at their site of capture. Sex and body measurements are reported in the supplemental metadata file (File S1). Body measurements were not included in our analysis as this generally reflected age (which was analyzed). Sex was only able to be determined for a subset of sexually mature adults and was therefore not included in our analysis, as the usable sample size was significantly less than our total number of samples collected.

### Nucleic acid isolation

DNA from swabs was extracted using a DNeasy PowerSoil Pro kit (QIAGEN, Venlo, the Netherlands). The extraction protocol included minor modifications to the manufacturer’s protocol, to include incubation of the swab in C1 buffer at 65°C for 10 min at 40 rpm, bead-beating for 90 s on a Mini-Beadbeater-96 (Biospec, Bartlesville, OK), and prior to the final elution having C6 warmed to 60°C and added to samples for a 5 min incubation. Negative extraction controls and whole-cell positive control community standards (Zymo Research, Irvine, CA, USA: Cat No: 6300) were also processed here alongside our samples to monitor for contamination and extraction efficacy, respectively.

### 16S rRNA gene sequencing

Extracted DNA from turtle samples, negative controls (environmental, extraction blanks, and PCR no-template controls), and positive control community standards (Zymo Research, Irvine, CA, USA: Cat Nos: 6300 and 6305) were prepared for 16S rRNA gene sequencing using barcoded primers 515F-Y and 939R (V4-V5 region) and sequenced on an Illumina MiSeq with v3 2 × 300 chemistry using the methods described in a previous study by Bornbusch et al. (23). Negative controls yielded very low read counts compared to experimental and positive controls (<150 reads vs. tens of thousands of reads).

### Data processing and statistical analysis

Raw sequencing data in fastq.gz format were first preprocessed with dada2 (24; Supplemental Jupyter Notebook: Dada2). Likely contaminants were identified and removed using the decontam package in R (25; combined frequency and prevalence method with DNA concentration and control/sample indicator), along with ASVs matching Zymo positive-control taxa. Zymo-positive controls recovered the expected genera at the expected abundances. All control samples were excluded from downstream analyses. Singletons and non-target organelle reads from chloroplasts were also removed during preprocessing. These cleanup steps resulted in a total of 1,227,148 reads (7,251 unique taxa and 80 samples) being reduced to 1,084,608 reads (2,897 unique taxa and 64 samples; Supplemental Jupyter Notebook: Preprocessing). All remaining samples with more than 2,500 reads were retained for analysis by pruning. The minimum sequencing depth among these remaining samples was 4,714 reads. All remaining samples were then rarefied to an even depth of 4,714 reads/sample using the rarefy_even_depth function in phyloseq and following results from rarefaction richness curve analysis (Fig. S1). Rarefaction resulted in a final total of 282,840 reads (2,805 unique taxa with 60 samples). These preprocessed, contaminant removed, pruned, and rarefied data were then subject to statistical analysis and visualization with a variety of packages, including phyloseq (26), vegan (27), and ggplot2 (28). Assumptions of normality were confirmed using histograms, Shapiro-Wilk tests, and Levene’s test (Supplemental Jupyter Notebook: Analyses). Files used in the statistical analysis included the metadata file, corresponding feature and taxonomy tables, and a fasta file of all DNA sequences used (File S1, S2 and S3, respectively). An α = 0.05 was used for all analyses.

Next, we examined microbial community alpha-diversity (ASV richness) as a function of different factor interactions (habitat site, body site, age, and pitting stage). Different linear mixed-effects models were examined for best fit using Akaike information criterion (AIC) and Bayesian information criterion (BIC) (*n* = 14 models, Supplemental Jupyter Notebook: Analyses). These models allowed us to determine if the factors of interest explained the bacterial communities among different groups of turtles. From these results, it was determined the most parsimonious model included all terms without interactions and also a random effect to account for non-independence of samples collected from the same individual turtle (S.obs_rare ~body_site + pits + site + age + (1 | marked.turtle)). Post hoc comparisons were then conducted using Tukey-adjusted estimated marginal means to determine significant differences among factors.

Following alpha-diversity analysis, beta-diversity was examined. The same rarefied ASVs were also used in beta-diversity statistical analyses. The four factors of interest (body site, habitat site, age, and shell algae status) were examined using both the Bray-Curtis and Jaccard dissimilarity measures for beta-diversity using permutational analysis of variance (PERMANOVA). To account for non-independence of samples from the same turtle, we completed the analysis with permutations constrained by individual (strata = marked.turtle). Model selection was determined by adonis2 using a combination of biological interest, best model R^2^, and parsimony to avoid excess complexity and overfitting. Statistical differences for these metrics were visualized using principal coordinate analysis.

Differentially abundant taxa were visualized using the DESeq2 package (29). The analysis was conducted on a phyloseq object containing raw ASV count data. Relative abundance transformations were applied to the count data to facilitate the visualization via a heatmap. Normalization was conducted using DESeq2’s default method. The differential abundance results for visualization identified taxa considered significantly differentially abundant using the following parameters: false discovery rate-adjusted *P* < 0.05 and a log2 fold change >1 or <−1.

To complement the DESeq2 findings and control for pseudoreplication due to repeated sampling of individual turtles, a statistically rigorous analysis was conducted using ANCOM-BC2 (30), a method designed to handle random effects in complex data sets. The ANCOM-BC2 analysis was performed by sequentially setting each body site as the reference group and comparing it against the others. This allowed for the identification of taxa that were significantly enriched or depleted in each body site relative to the other body sites. Multiple runs of ANCOM-BC2 were used to account for the inability of a single run to carry out all pairwise comparisons and corresponding summary statistics, in part due to the challenges with the sparsity of our data matrices. This resulted in four separate pairwise comparison tables, one for each body site as reference (cloaca, skin, plastron, and carapace). Each table contains a subset of overlapping pairwise comparisons with the other tables, but the results are generally the same, excepting differences in sign due to the direction of the pairwise comparisons and some minor numeric differences due to random effects in each model run. All tables, and also the top five log-fold changed taxa table, are included for transparency. The analysis was conducted using standard parameters (Supplementary Jupyter Notebook: Analyses). Briefly, a phyloseq object was used in conjunction with a model using both fixed effects (body site, habitat site, and pitting stage) and a random effect (individual turtle ID) to account for pseudoreplication. A grouping argument for body site was also included, as this was the factor of interest based on alpha and beta-diversity analyses.

## RESULTS

### Taxonomic abundances

The most commonly represented phyla across samples included Proteobacteria, Bacteroidota, Firmicutes, Actinobacteriota, and Cyanobacteria (Fig. S2). Proteobacteria and Firmicutes were among the most represented phyla across all sample types. Across all samples, 45 unique phyla were identified. Representation of other taxonomic levels, including genus and species, is available in File S2.

### Alpha-diversity

**Fig 2:**
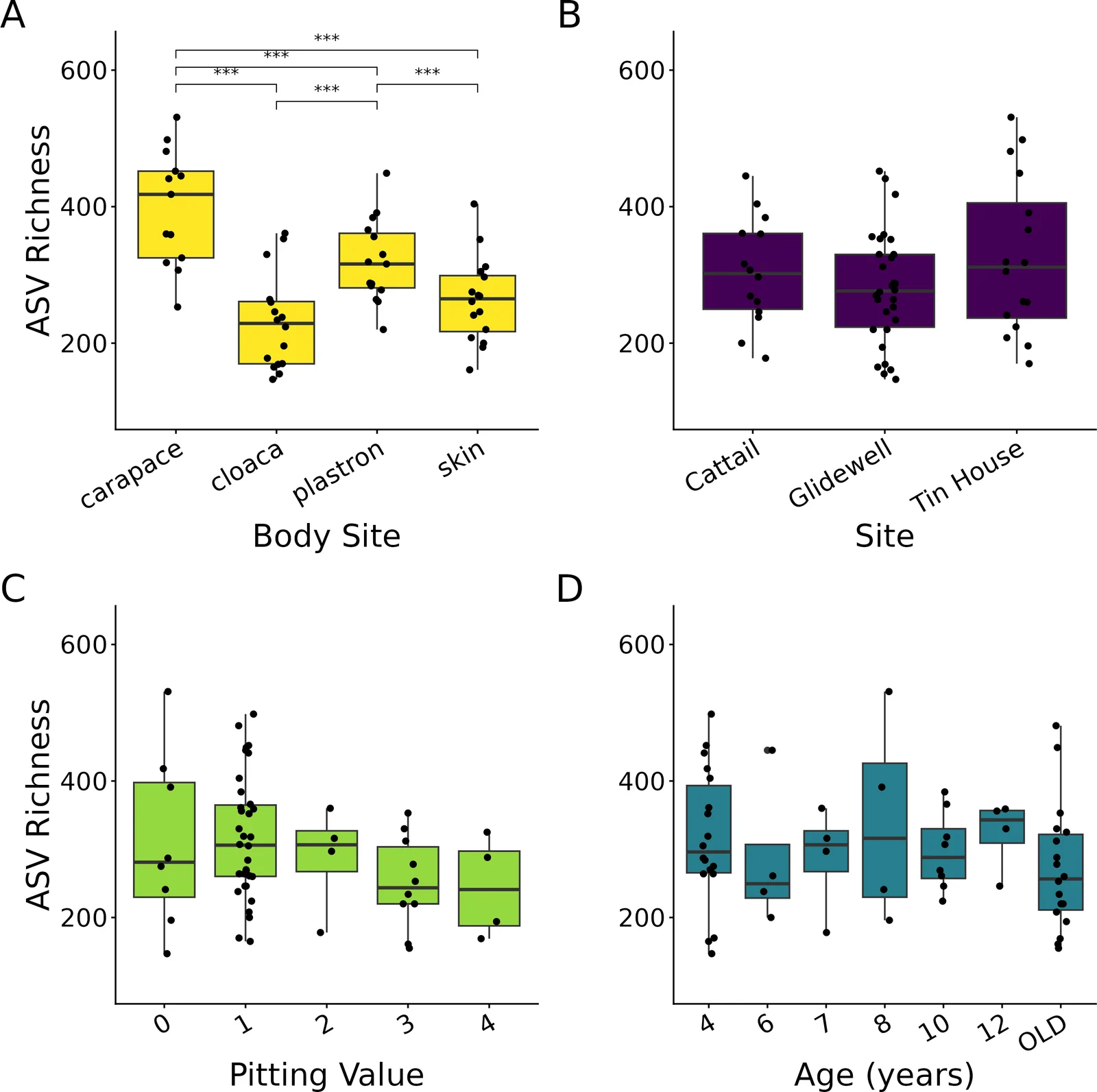
Boxplots with jittered data points of bacterial ASV richness by (**A**) turtle body site, (**B**) habitat site, (**C**) turtle shell pitting stage, and (**D**) turtle age. Tukey’s pairwise tests of estimated marginal means indicate significant pairwise comparisons between a subset of turtle body sites (*p* < 0.05 indicated by ***). The other factors of interest (**B–D**) demonstrated no omnibus significance.

Amplicon sequence variant (ASV) richness (alpha-diversity) was first examined using linear mixed-effects models with different combinations of the four factors of interest: body site (carapace, cloaca, plastron, and skin), habitat site (Cattail Pond [permanent pond], Glidewell [permanent pond], and Tin House [ephemeral pond]), age (4, 6, 7, 8, 10, 12, and “older”), and shell pitting stage (0, 1, 2, 3, and 4). A term for random effects by turtle was also included (1 | marked.turtle) to account for pseudoreplication of multiple swabs from the same individual turtle. The best fitting model based on AIC and BIC included body site, age, and pitting stage, without interaction effects (S.obs_rare ~body_site +pits + site +age + (1 | marked.turtle)). A more complex model with an interaction between body site and habitat site was also considered, but this did not significantly improve the model fit (ΔAIC = +2.5, *P* = 0.15); hence, the model without interactions was used. This model also indicated that observed microbial richness (S.obs_rare) varied significantly among body sites (*P* < 0.05), but not with site, age, or shell pitting stage (*P* > 0.05). Tukey’s pairwise tests were then performed using estimated marginal means, which showed that the carapace showed the highest ASV richness and differed from all other body sites (Supplemental Jupyter Notebook: Analyses). The plastron also had high ASV richness being significantly higher than the cloaca and skin (Fig. 2; *P* < 0.05). Significant differences were not seen between skin and cloaca (*P* > 0.05).

### Beta-diversity

**Fig 3:**
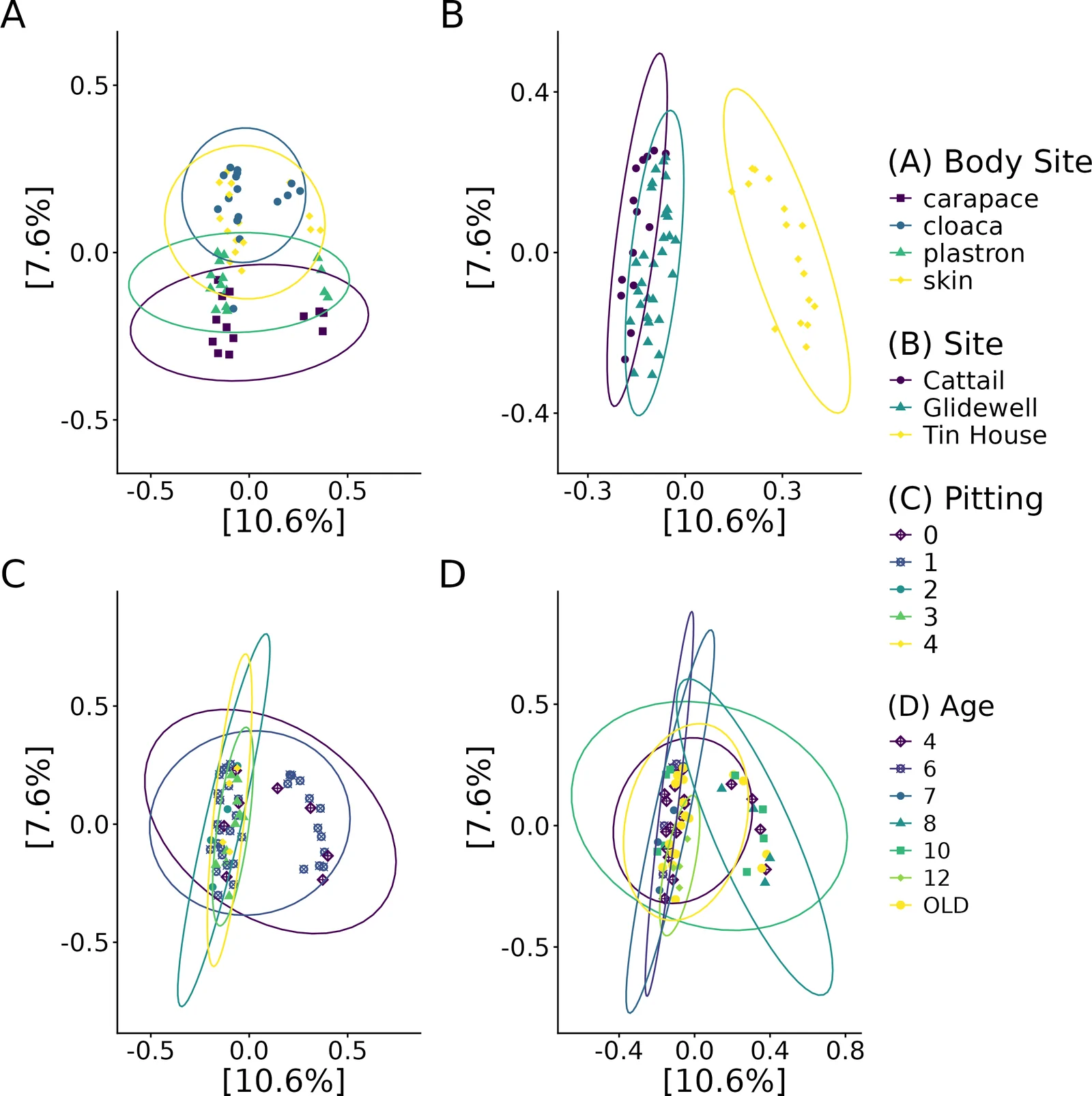
Principal coordinate analyses of bacterial community beta diversity using the Jaccard dissimilarity metrics. Data groupings are given by (**A**) body site, (**B**) site, (**C**) pitting stage, and (**D**) age. Data ellipses show a 95% confidence and assume a multivariate t-distribution. Color subgroups by factor are given in the corresponding legends.

Between-community beta diversity was also examined using the four factors of interest: body site, habitat site, age, and shell pitting stage using Jaccard and Bray-Curtis measures. For the Jaccard dissimilarity measure (Fig. 3), a model containing all main effects (body site, site, age, and pitting stage) was selected, while accounting for pseudoreplication by including individual turtle ID as a strata factor. The overall PERMANOVA model explained ca. 37% of the variation in microbial communities (R² = 0.37, F = 1.88, *P* = 0.001).

Permutational tests by term indicated significant explanatory power for all of the included main effects with observed Jaccard beta diversity as the response: body site (R² = 0.089, F = 2.11, *P* = 0.001), site (R² = 0.063, F = 2.26, *P* = 0.001), pitting stage (R² = 0.058, F = 1.38, *P* = 0.001), and age (R² = 0.091, F = 1.30, *P* = 0.001). Pairwise PERMANOVA tests with stratification by turtle ID (to account for pseudoreplication) showed that bacterial community composition differed significantly among body sites (all pairwise comparisons *P* (adjusted) <0.01; Supplemental Jupyter Notebook: Analyses). However, no significant pairwise differences were detected among habitat sites, pitting stage, or age after adjusting for multiple testing.

Tests for homogeneity of group dispersions confirmed that multivariate dispersion differed significantly among habitat site, pitting stage, and age (*P* < 0.05; Supplemental Jupyter Notebook: Analyses), but not among body sites (*P* > 0.05). This indicates that the observed diversity differences in habitat site, pitting stage, and age categories may reflect within-group heterogeneity. However, the non-significant results for beta-dispersion in body sites indicated that the significant differences observed reflect true microbial community differences among body sites.

For the Bray-Curtis dissimilarity measure (Fig. S3), the results were similar to those for Jaccard, and the same model was used containing all main effects (body site, site, age, and pitting stage), while accounting for pseudoreplication by including individual turtle ID as a strata factor. The overall PERMANOVA model explained approximately 58% of the variation in microbial communities (R² = 0.58, F = 4.52, *P* = 0.001).

Permutational tests by term indicated significant explanatory power for all of the included main effects with observed Bray-Curtis beta-diversity as the response: body site (R² = 0.16, F = 5.80, *P* = 0.001), site (R² = 0.11, F = 5.84, *P* = 0.001), pitting stage (R² = 0.068, F = 2.47, *P* = 0.001), and age (R² = 0.097, F = 2.11, *P* = 0.001). Pairwise PERMANOVA tests with stratification by individual turtle ID showed that bacterial community composition differed significantly among body sites (all pairwise comparisons *P* [adjusted] < 0.001; Supplemental Jupyter Notebook: Analyses). However, and the same as Jaccard dissimilarity, no significant pairwise differences were detected among habitat sites, pitting stage, or age after adjusting for multiple testing.

Tests for homogeneity of group dispersions confirmed that multivariate dispersion differed significantly among body site, habitat site, pitting stage, and age (*P* < 0.05; Supplemental Jupyter Notebook: Analyses), with body sites close to our significance threshold (*P* = 0.023). This indicates that the observed diversity differences in habitat site, pitting stage, and age categories may reflect within-group heterogeneity. Additionally, the weakly significant beta-dispersion in body sites indicated that the significant differences observed among body sites are in part attributable to within-group heterogeneity.

### Differential abundance visualization and analysis among bacterial communities

**Fig 4:**
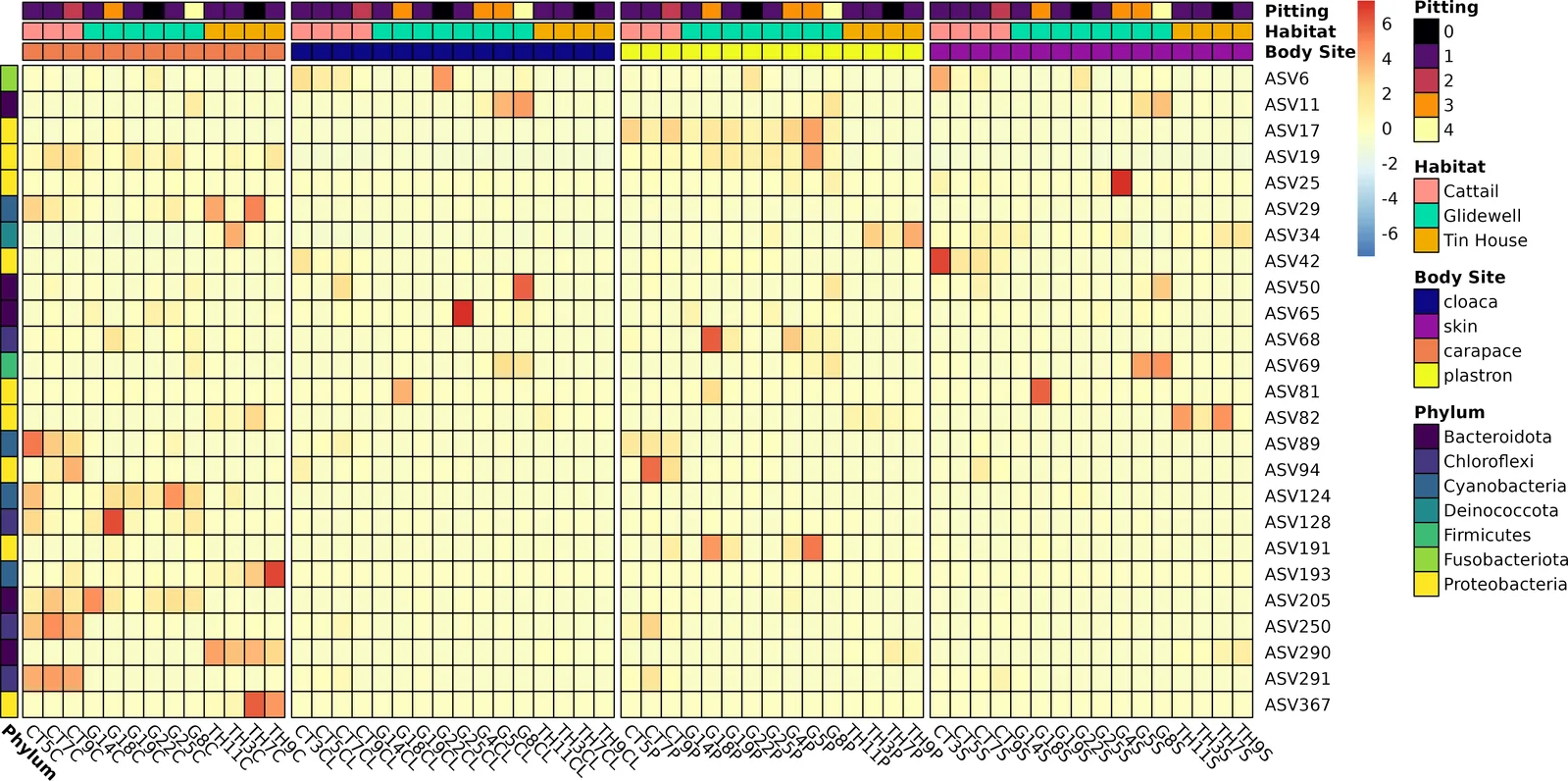
Top 25 differentially abundant ASVs across all modeled factors. Rows are ordered by decreasing ASV abundance from top to bottom, with ASV ID given on the right and the seven key phyla indicated by color bars on the left. Columns are grouped by body site, with habitat and pitting also shown. Turtle samples missing from the bottom of the figure lacked any of the top 25 ASVs.

A heatmap was used to visualize relative abundances of the top 25 bacterial taxa across all samples containing these top 25 differentially abundant taxa (Fig. 4). Samples were categorized by habitat site, pitting stage, and body site (age was excluded due to low replicate numbers and complexity in visualization). These results indicated clear clustering by body site factor indicating enrichment of seven discrete phyla. The carapace in particular showed an overabundance of ASVs representing Cyanobacteria and Chloroflexi, whereas the plastron exhibited an overabundance of ASVs representing Proteobacteria.

Following visualization indicating body site groupings, a differential abundance analysis with ANCOM-BC2 was conducted, controlling for pseudoreplication (Table S2 and File S4). The analysis identified several bacterial taxa with both qualitative and quantitative significant enrichment in pairwise comparisons by body site, particularly with the shell, carapace, and plastron, showing greater differentiation from skin and cloaca (Fig. 4). *Spirosoma*, a gram-negative and facultative anaerobic genus (31), was more prevalent on the skin when compared to cloaca, carapace, and plastron, indicating that this is an important skin-associated genus in *K. flavescens*. In the cloaca, the T34 family of the Burkholderiales was also qualitatively enriched as compared to the carapace, plastron, and skin. Also, in the cloaca, significant reduction of the genus *Rubellimicrobium* (q < 0.05) was observed when compared to the plastron and carapace. The gram-negative anaerobic genus *Paludibacter* (32) was also observed to be significantly enriched in the cloaca as compared to skin (q < 0.05) and also qualitatively more abundant in the cloaca versus plastron (Table S2). Cyanobacteria was seen to be significantly enriched in the carapace as compared to the plastron (q < 0.05). These findings indicate that various bacterial taxa with diverse functions are significantly enriched or depleted among body sites.

## DISCUSSION

The results of this study indicate both significant bacterial community diversity differences and specific differential taxa abundance among body sites in the Yellow Mud Turtle, *K. flavescens*. Important bacterial community effects were also observed due to habitat site, pitting stage, and age. These results indicate important nuances in microbial community diversity among factors in *K. flavescens*. Also, these results generally support similar, but limited, studies in other species of turtles and have important implications for future turtle microbiome studies.

Most body sites on *K. flavescens* significantly differed in bacterial ASV richness, particularly the carapace, which had the highest ASV richness compared to the cloaca, plastron, and skin, followed by the plastron having higher richness than the cloaca. McKnight et al. (9) similarly found microbial community diversity in Krefft’s River Turtle (*Emydura macquarii krefftii*) varied between the shell, buccal cavity, and head, with the buccal cavity in that study exhibiting lower richness than the other body sites. In the marine Loggerhead Sea Turtle (*Caretta caretta*), significantly lower bacterial alpha diversity in the cloaca was observed compared to oral and carapace regions (12), suggesting that the cloaca may generally be a low diversity body site in turtles. In the relatively large *C. caretta* carapace, differences in bacterial richness were observed between the anterior and posterior scutes (33), thereby suggesting that differences in bacterial richness among body sites are a general feature of turtles across their environments. Future studies examining turtle microbiomes might consider dropping skin sampling if already including cloaca, carapace, and plastron, especially if greater sampling efficiency or cost reduction is desired. Specifically, in *K. flavescens*, skin richness did not exhibit significant pairwise differences with the cloaca, with which it also had overlapping taxa. This difference may be due to the more direct connectedness of the external interface of the cloaca, which gradates into skin, while having more physical separation from the carapace and plastron. While not under investigation in this study, the degree of microenvironment connectedness in turtle body sites is not well known and may determine whether body site differences should be treated as categorically distinct or as gradients of community change. This could include examining microbial communities over the body of a single individual turtle under higher resolution (more sampling sites) in future work.

For beta-diversity comparisons, significant differences were seen in all body site pairwise comparisons. This result was observed in both the Jaccard and Bray-Curtis measures, indicating that significant community differences occur among total unique taxa and also among unique taxa with abundance taken into account. Of the four body sites examined, cloacal sampling remains an important non-invasive mode of sampling for turtle-microbiome evolutionary studies, as it contains the microbiome component most likely to be transmitted during egg laying, thereby encompassing intergenerational inheritance or transfer. A strong probability of microbial intergenerational transmission between the egg and cloaca has been previously reported in the Chinese Pond Turtle (*Mauremys reevesii*; 34) and the Yellow-spotted Amazon River Turtle (*Podocnemis unifilis*; 35). While understanding any potential sex differences may therefore be important due to physiological differences in sexually mature adults, sex was not included as a factor in our analyses because of the limited sample size of individuals with phenotypically identifiable sex. Many of the younger turtles sampled lacked clear external characteristics used for sex determination. Some of the unaccounted for community variation observed in our study may therefore be due to sex differences, which future studies with larger sample sizes could further evaluate.

Interestingly, while habitat site, pitting stage, and age showed significance as overall terms in our beta-diversity models, they did not exhibit any significant pairwise differences after adjusting for multiple testing. Although individual factors explained some independent proportions of beta-diversity variation (partial R^2^ < 0.20), these values represent typical ecological community complexity where multiple weaker factors are operating together, resulting in larger emergent effects. While these factors exhibited statistical significance, most showed modest effect sizes (partial R^2^ < 0.10), and body site explained the largest proportion (partial R^2^ = 0.16 for Bray-Curtis beta-diversity), representing our most biologically meaningful result. Additionally, these results may also be a reflection, in part, of low sample size, which will require future study to fully elucidate.

Differences in bacterial community composition among habitat sites are partially supported by previous work with False Map Turtles (*Graptemys pseudogeographica*) that showed differences in cloacal microbiome diversity across habitat type in the Lower Missouri River at both term and pairwise levels of analysis (11). Host species traits may also play a role in regulating the number of bacterial taxa that can colonize a specific body site, while habitat-level characteristics may influence the environmental pool of microorganisms that can colonize that body site. For example, Parks et al. (36) compared carapace microbiomes among six turtle species and three sites, with species having a larger role in determining microbial community diversity than differing habitat sites. The natural history of *K. flavescens* is markedly different from any other turtle where microbiome data have been published. Their unique ecology, where individuals are utilizing aquatic environments 2–12 weeks each year, coupled with their 9-month brumation in an arid landscape, makes direct comparisons of microbiome results difficult with other taxa and across other habitats.

Our study did not collect standardized water quality measurements for each pond habitat. These shallow ponds have strong spatial and temporal heterogeneity among environmental factors; hence, point measurements would not reliably represent the environmental conditions turtles experience. As we cannot link microbiome variation with environmental gradients, we acknowledge that this is an important limitation. Future work should incorporate a sampling design with a robust environmental component to better understand how habitat heterogeneity influences turtle microbiome variation.

Important findings with implications for turtle health were also observed in our differential abundance analysis. Cyanobacteria were significantly more abundant on the carapace than plastron. In particular, Cyanobacteria associated with the turtle carapace, but not the plastron, are suggestive of a niche for photosynthetic microbes due to the increased exposure of the carapace to sunlight as compared to other parts of the turtle body. Interestingly, this association has previously been studied in this population with similar results. Christiansen et al. (14) identified Cyanobacteria associated with carapace pitting in at least one sample during their investigation into causal mechanisms of an algae-mediated shell disease. Significantly higher abundance of Cyanobacteria was also associated with shell lesions in pond turtles (*Actinemys* sp.) (37). This work also suggested filamentous algae, such as *Arnoldiella chelonum,* have a role in the pitting condition, resulting in layers of keratin shed from the carapace (14). However, the relation between carapacial pitting, Cyanobacteria, and algae is at this point unclear. The microbiome associated with the turtle carapace, as observed in this study, might also be structured around the presence of Cyanobacteria. The presence of Chlorflexi, some of which are also photosynthetic, may also lend support to conceptualizing the turtle carapace community as a guild using the resource of sunlight (38). Why the turtle may provide or tolerate a niche for Cyanobacteria and algae to grow, and how the turtle and its associated carapacial microbiome could possibly benefit from such a relationship should be examined in future investigations. Additionally, the possible application of microbial guild theories presents an exciting conceptual paradigm for future work on the ecology and evolution of turtle microbiomes, particularly in the evolution of the shell. In addition to the differences observed among broad taxonomic groups, there were specific bacterial genera that were observed to be differentially abundant in the pairwise differential abundance analysis. In particular, the bacteria of the genus *Paludibacter* are of interest due to their significant enrichment in the cloaca as compared to other body sites. While not known to be specifically passed between generations during egg laying, this possibility should be examined in future work.

Finally, while this study provides important baseline data on *K. flavescens* microbiomes across habitats and body sites, our sample size (*n* = 16 turtles with four swabs per turtle; *n* = 64 turtle swabs) limits statistical power to detect weaker effects and subtle patterns that may be of interest. Although individual turtles were selected to broadly represent three habitats and age gradients, a more robust sampling effort in future work may reveal subtle effects not seen in this study. Additionally, factors including sex, seasonal variation, and microenvironment gradients were not explored due to the logistical and cost limitations, resulting in the current sample size. These limitations highlight the importance of our findings to guide more comprehensive future work.

## Supplementary Material

Reviewer comments

## Data Availability

All raw 16S rRNA gene sequencing files used in this study are available at the NCBI Sequence Read Archive as BioProject PRJNA1154396, and all analysis files are provided as supplement. Jupyter files are available in GitHub at https://github.com/kvasir7/Jupyter_Files_Yellow_Mud_Turtle.

## AFTER EPUB

*[This article was published on 20 July 2026 with an error in the References. The error was corrected in the current version, posted on 30 July 2026.]*

## SUPPLEMENTAL MATERIAL

Figure S1 spectrum.03664-25-s0001.tiff Read counts for rarefaction.

Figure S2 spectrum.03664-25-s0002.tiff Abundance barplots.

Figure S3 spectrum.03664-25-s0003.tiff Beta diversity plots, Bray-Curtis.

Table S1 spectrum.03664-25-s0004.xlsx Sample distributions by factor.

Table S2 spectrum.03664-25-s0005.xlsx Ancom-bc2 results.

File S1 spectrum.03664-25-s0006.xlsx Metadata file for study.

File S2 spectrum.03664-25-s0007.xlsx Taxonomy file that includes taxonomic IDs for all ASVs present.

File S3 spectrum.03664-25-s0008.FASTA This file contains the DNA sequences in fasta format for all ASVs.

File S4 spectrum.03664-25-s0009.xlsx This file contains multiple sheets of pairwise comparison results from ANCOM-BC2 analysis.

Supplemental legends spectrum.03664-25-s0010.docx Legends for Files S1 to S4, Figures S1 to S3, and Tables S1 and S2.

OPEN PEER REVIEW reviewer-comments.pdf An accounting of the reviewer comments and feedback.

ASM does not own the copyrights to Supplemental Material that may be linked to, or accessed through, an article. The authors have granted ASM a non-exclusive, world-wide license to publish the Supplemental Material files. Please contact the corresponding author directly for reuse.
