# Supplementary material for: Microbiome varies by body site in the yellow mud turtle, Kinosternon flavescens, from the Chihuahuan Desert
Source: Microbiol Spectr. 2026 Jul 20;14(9):e03664-25. doi: 10.1128/spectrum.03664-25 (PMC13532361; doi:10.1128/spectrum.03664-25)
Supplement: Supplementary file 1 [file reviewer-comments.pdf]

AMERICAN SOCIETY FOR MICROBIOLOGY | Microbiology Spectrum

# Microbiome Varies by Body Site in the Yellow Mud Turtle, *Kinosternon flavescens,* from the Chihuahuan Desert

Joseph Madison, Drew Davis, Benjamin Genter, Travis LaDuc, and Carly Muletz Wolz

*Corresponding Author(s): Joseph Madison, Xavier University of Louisiana*



## Review Timeline:

| | |
|---|---|
| Submission Date: | November 13, 2025 |
| Editorial Decision: | January 2, 2026 |
| Revision Received: | April 3, 2026 |
| Accepted: | April 23, 2026 |

*Editor: Samara Mattiello*

*Reviewer(s): Disclosure of reviewer identity is with reference to reviewer comments included in decision letter(s). The following individuals involved in review of your submission have agreed to reveal their identity: Yuchao Zhao (Reviewer #1); Jian Yang (Reviewer #2)*

## Transaction Report:

(Note: With the exception of the correction of typographical or spelling errors that could be a source of ambiguity, letters and reports are not edited. The original formatting of letters and referee reports may not be reflected in this compilation.)

**DOI: https://doi.org/10.1128/spectrum.03664-25**

Re: Spectrum03664-25 (Microbiome Varies by Body Site in the Yellow Mud Turtle, *Kinosternon flavescens,* from the Chihuahuan Desert)

Dear Dr. Joseph D Madison:

Thank you for the privilege of reviewing your work. Below you will find my comments, instructions from the Spectrum editorial office, and the reviewer comments.

Please return the manuscript within 60 days; if you cannot complete the modification within this time period, please contact me. If you do not wish to modify the manuscript and prefer to submit it to another journal, notify me immediately so that the manuscript may be formally withdrawn from consideration by Spectrum.

**Revision Guidelines**
To submit your modified manuscript, log into the submission site at https://spectrum.msubmit.net/cgi-bin/main.plex. Go to Author Tasks and click the appropriate manuscript title to begin. The information you entered when you first submitted the paper will be displayed; update this as necessary. Note the following requirements:

• Upload point-by-point responses to the issues raised by the reviewers in a file named "Response to Reviewers," NOT in your cover letter.
• Upload a compare copy of the manuscript (without figures) as a "Marked-Up Manuscript" file.
• Upload a clean .DOC/.DOCX version of the revised manuscript and remove the previous version.
• Each figure must be uploaded as a separate, editable, high-resolution file (TIFF or EPS preferred), and any multipanel figures must be assembled into one file.
• Any supplemental material intended for posting by ASM should be uploaded with their legends separate from the main manuscript. You can combine all supplemental material into one file (preferred) or split it into a maximum of 10 files with all associated legends included.

For complete guidelines on revision requirements, see our Submission and Review Process webpage. Submission of a paper that does not conform to guidelines may delay acceptance of your manuscript.

**Data availability:** ASM policy requires that data be available to the public upon online posting of the article, so please verify all links to sequence records, if present, and make sure that each number retrieves the full record of the data. If a new accession number is not linked or a link is broken, provide Spectrum production staff with the correct URL for the record. If the accession numbers for new data are not publicly accessible before the expected online posting of the article, publication may be delayed; please contact production staff (Spectrum@asmusa.org) immediately with the expected release date.

**Publication Fees:** For information on publication fees and which article types are subject to charges, visit ourwebsite. If your manuscript is accepted for publication and any fees apply, you will be contacted separately about payment during the production process; please follow the instructions in that e-mail. Arrangements for payment must be made before your article is published.

**ASM Membership:** Corresponding authors may join or renew ASM membership to obtain discounts on publication fees. Need to upgrade your membership level? Please contact Customer Service at Service@asmusa.org.

The ASM Journals program strives for constant improvement in our submission and publication process. Please tell us how we can improve your experience by taking this quick Author Survey.

Thank you for submitting your paper to Spectrum.

Sincerely,
Samara Mattiello
Editor
Microbiology Spectrum

Reviewer #1 (Comments for the Author):

This article represents a moderate-quality academic contribution in the field of turtle microbiome research. Its core value lies in establishing a basic understanding of the association between the microbiome of the yellow mud turtle and multiple factors, and in raising scientific questions worthy of further investigation. If improvements are made regarding sample representativeness, functional annotation supplementation, and detail standardization, thereby further enhancing the reliability of the results and the

depth of mechanism interpretation, the academic impact and application value of this study will be significantly increased. It is fully capable of being published in relevant professional journals and can provide an important reference for research on reptile microbiomes and host-environment interactions.

1.Line 86, "Previously, differences in body parts and habitats of the same species were not studied simultaneously, and reptile microbiome research was not comprehensively reviewed." It is recommended to supplement the description of the search scope.

2.Line 90, "a potential health issue in turtles with implications to conservation efforts." Please provide a detailed description of the incidence rate, the overall impact, and other relevant information, and supplement the background information with research progress.

3.Line 251,Habitat may influence the environmental pool for microbial colonization, but this was not analyzed in relation to the environmental differences between temporary and permanent ponds in this study. It is recommended to supplement with habitat environmental parameter data, such as pH, dissolved oxygen, and nutrients, and to investigate the mechanisms by linking them to changes in the microbiome.

4.Line 249,The discussion of research limitations is not comprehensive; it is recommended to add a thorough analysis of the limitations and propose targeted directions for future research.

5.Line 315, There are a total of 72 turtles, but only 16 are selected for the study. Please specify the selection criteria in detail and clarify the representativeness of the 16 turtles in the overall population.

6.Line 327-329，Environmental control swabs and air negative controls were used, but the microbial composition analysis of the control samples was not presented in the results, nor was it explained how the control data were used to rule out sample contamination. Can the sequencing results of the control samples support the reliability of the experimental samples? Is it necessary to supplement the analysis with the microbial overlap between the controls and the experimental samples?

7.Line 337-338, Age was determined by counting growth rings on the pectoral (plastral) scute. Add some references.

8.The chart labels are unclear. It is recommended to check and correct any spelling errors, and if necessary, add indicators of statistical significance in the chart.

9.The paper points out that there is no significant difference in ASV richness between the skin and cloaca, and their taxa overlap, but the physiological microenvironments of the two are significantly different. The paper does not explain the ecological mechanism behind this taxonomic overlap. Could you tell me if there are any related hypotheses or plans for further validation?

10.Inconsistent reference formatting: some references lack author names, publication years, or journal information, and some author name abbreviations are inconsistent. It is recommended to standardize the references according to the journal's format, fill in the missing information, and unify the formatting.

Reviewer #2 (Comments for the Author):

This manuscript systematically investigates the differences in microbial communities across various body sites of the Yellow Mud Turtle (Kinosternon flavescens), while also assessing the effects of habitat, age, and severity of shell disease. Using 16S rRNA sequencing, linear mixed-effects models, and PERMANOVA analyses, this study presents clear conclusions with significant ecological implications, particularly demonstrating innovation in linking variations among body sites with associations to Cyanobacteria. However, there are some flaws in experimental design and statistics, and some interpretations appear to extend beyond the direct support of the data. The detailed comments are as follows:

1. Lines 37-38: The abstract claims that bacterial community differences exist among habitat types. However, the results section (lines 146-147) indicates that no significant pairwise differences were detected among habitat sites after correcting for multiple testing. In the discussion (lines 247-262), the authors reference previous studies to support the effects of habitat. This may lead readers to overestimate the strength of evidence derived from this dataset. It is recommended that the conclusive statement in the abstract be revised to more accurately reflect the statistical evidence presented in the results.

2. Figure 3 (Heatmap): The clustering of samples in Figure 3 is overly dense, reducing readability. Additionally, although different phyla are color-coded, bacterial taxa from the same phylum are scattered throughout the rows, making intuitive interpretation of patterns at the phylum level difficult. To enhance the clarity and informational value of this figure, it is recommended to: (1) simplify or optimize the display of sample clustering, (2) group taxa from the same phylum adjacently, and (3) focus the main-text figures on key comparisons for improving readability.

3. Lines 249-250: As the authors indicated in the discussion, the relatively small sample size may have influenced some statistical outcomes. Nevertheless, the modest independent explanatory power (partial $R^2$) of the tested factors-most explaining less than 10% of the variation-suggests limited individual ecological effects. A brief discussion on the biological interpretation of these effect sizes, along with a simple visualization (e.g., variance partitioning), would enhance the clarity and ecological context of the β-diversity results.

4. Lines 222-262: The discussion cites studies of other turtle species to support established patterns but does not sufficiently engage in a deeper comparison that incorporates the unique ecological traits of the study species (e.g., its strong terrestrial

habits and arid habitat). It is recommended to utilize these comparisons to further elucidate the distinctive findings of this study. For example, could the patterns of body-site microbiome differentiation observing in Kinosternon flavescens, in comparison to the more aquatic species referenced, be linked to its unique life-history features such as terrestrial activity and exposure to dry conditions?

5. Lines 263-265: The text indicates that cyanobacterial abundance differs among groups categorized by shell pitting severity. However, this result only states that "a difference exists" without clarifying the specific pattern of change (e.g., whether abundance increases with worsening pitting). The discussion is too speculative regarding a functional association, such as photosynthetic commensalism, thus lacks clear trend data support.

6. Lines 335-339: The study includes age (7 groups) and shell pitting stage (5 groups) as factors. However, the number of individual turtles per specific age group or pitting stage is not provided. Given the total sample size of only 16 turtles, it is likely that some groups contain very few samples, which can substantially reduce statistical power and affect the reliability of the results. It is suggested that this information be included (e.g., in a supplementary table), and the limitation noted in the discussion to assist readers in interpreting these results.

7. Lines 120-175, 214-221: Although the authors reported several statistically significant findings ($p < 0.05$), it would strengthen the discussion to differentiate more clearly between statistical and biological significance. Clarifying which effects are small but statistically detectable versus ecologically meaningful will improve the interpretive balance of the results.

8. Lines 32-38, 98-99, 247-248, 340-343: Although age was included as a factor, sex was not analyzed due to small sample size. Given that sexual maturity can influence microbiome composition, especially in cloacal and skin regions, it would be valuable to briefly discuss how the absence of sex data might limit the interpretation of host-associated microbial differences.

9. Lines 496-498: The formatting of reference years is needed.

**Re: Response to Reviewers for revisions to Spectrum03664-25 (Microbiome Varies by Body Site in the Yellow Mud Turtle, *Kinosternon flavescens,* from the Chihuahuan Desert)**

Please find below our point-by-point response to reviewers. The responses (italicized) are given below reviewer comments and have line numbers referencing changes in the updated manuscript, where appropriate. Please note that the Materials and Methods section was moved to be immediately after the Introduction in the final revised draft (no markup), in-line with ASM Microbiology Spectrum formatting. Line number references given in our responses to reviewers reference the revised version that includes markup. The reference list has also been change from alphabetical to numerical, in order of appearance in text. Lastly, legends for all supplementary figure, files, and tables have been included in a separate document (as requested by editorial office).

**Reviewer #1 (Comments for the Author):**

This article represents a moderate-quality academic contribution in the field of turtle microbiome research. Its core value lies in establishing a basic understanding of the association between the microbiome of the yellow mud turtle and multiple factors, and in raising scientific questions worthy of further investigation. If improvements are made regarding sample representativeness, functional annotation supplementation, and detail standardization, thereby further enhancing the reliability of the results and the depth of mechanism interpretation, the academic impact and application value of this study will be significantly increased. It is fully capable of being published in relevant professional journals and can provide an important reference for research on reptile microbiomes and host-environment interactions.

1.Line 86, "Previously, differences in body parts and habitats of the same species were not studied simultaneously, and reptile microbiome research was not comprehensively reviewed." It is recommended to supplement the description of the search scope.

*We thank the reviewer for this suggestion. We have included an additional sentence to clarify our search scope, which was focused on turtle microbiomes. "However, we note that general reptile microbiome research was not comprehensively reviewed here." (Lines 91-92)*

2.Line 90, "a potential health issue in turtles with implications to conservation efforts." Please provide a detailed description of the incidence rate, the overall impact, and other relevant information, and supplement the background information with research progress.

*Prevalence and negative impact of this condition has been added in a new sentence following this comment. We have also added additional text in the introduction and discussion. (Lines 93-100; 320-324)*

3.Line 251,Habitat may influence the environmental pool for microbial colonization, but this was not analyzed in relation to the environmental differences between temporary and permanent ponds in this study. It is recommended to supplement with habitat environmental parameter data, such as pH, dissolved oxygen, and nutrients, and to investigate the mechanisms by linking them to changes in the microbiome.

*We appreciate the reviewer's suggestion to incorporate water quality data for the ponds. However, we did not collect standardized environmental measurements for this study. This was due both to logistical*

*constraints and also because these ponds are shallow with strong spatial and temporal heterogeneity among temperature, dissolved oxygen, nutrients, etc. Point measurements would therefore not reliably represent the environmental conditions that the turtles would experience. To investigate these dynamics as suggested would require a sampling design beyond the scope of the present work. We now explicitly acknowledge this as a limitation in the discussion and have added language to make this clear, and we also suggest the inclusion of such questions and related sampling design in future work. (Line 300-309)*

4.Line 249,The discussion of research limitations is not comprehensive; it is recommended to add a thorough analysis of the limitations and propose targeted directions for future research.

*We thank the reviewer for this recommendation and have added additional text to our discussion section to make limitations clear. Specifically, we have added discussion related to lack of environmental data for ponds, and have also included an additional paragraph describing the limits of the sample sizes presented, and the ability of this work to motivate future more comprehensive studies (Lines 300-309; 341-349).*

5.Line 315, There are a total of 72 turtles, but only 16 are selected for the study. Please specify the selection criteria in detail and clarify the representativeness of the 16 turtles in the overall population.

*We thank the reviewer for raising this point. Of the 72 turtles captured over this field season, we selected 16 (with four swabs per individual) for additional microbiome sequencing to answer our questions based on logistical and cost constraints. We aimed to have a balanced representation of four individuals per pond which were randomly selected. We also added another four individuals from one pond to represent the broadest age range possible at that site. The selected individuals therefore span habitat sites, age classes, and also shell pitting status. We now clarify our selection rationale in the methods and note the sample size limitation in the discussion. (Lines 375-380; 341-349)*

6.Line 327-329,  Environmental control swabs and air negative controls were used, but the microbial composition analysis of the control samples was not presented in the results, nor was it explained how the control data were used to rule out sample contamination. Can the sequencing results of the control samples support the reliability of the experimental samples? Is it necessary to supplement the analysis with the microbial overlap between the controls and the experimental samples?

*We thank the reviewer for highlighting the importance of controls in our study. Environmental field control swabs (CPWC1, GWC1, and THWC1) and air negative control swabs (CPAC1, GAC1, and THAC1) were sequenced alongside turtle and pond samples and are presented in the Supplemental Jupyter Notebook (preprocessing). Air and lab negative control swabs yielded very low read counts compared to our experimental and field control samples (<150 reads in most air/lab negatives vs thousands to tens of thousands of reads in turtle and pond swabs), which is consistent with low-biomass background signal rather than true biological community signatures. We implemented a combined frequency and prevalence–based contaminant detection approach (using the decontam package in R) using both DNA concentration and a control/sample indicator, which identified 30 putative contaminant ASVs out of 2,937. These ASVs were removed, and all control samples were excluded from downstream analyses. Zymo positive controls recovered the expected genera at the expected abundances, confirming that library preparation and sequencing worked as expected. Overlap between negative controls and turtle samples was limited to low-abundance taxa flagged as contaminants during preprocessing. These steps ensure that the main community patterns reported in the manuscript are unlikely to be driven by contamination, and we have added additional detail to make these points clear in the Methods.*

*"Negative extraction controls and whole-cell positive control community standards (Zymo Research, Irvine, CA, USA: Cat No: 6300) were also processed here alongside our samples to monitor for contamination and extraction efficacy, respectively." (Lines 424-427)*

*"Likely contaminants were identified and removed using the decontam package in R (Davis et al., 2018; combined frequency and prevalence method with DNA concentration and control/sample indicator), along with ASVs matching Zymo positive-control taxa. Zymo positive controls recovered the expected genera at the expected abundances. All control samples were excluded from downstream analyses." (Lines 438-443)*

7.Line 337-338, Age was determined by counting growth rings on the pectoral (plastral) scute. Add some references.

*We have added a reference to this sentence (Iverson 1991).*

8.The chart labels are unclear. It is recommended to check and correct any spelling errors, and if necessary, add indicators of statistical significance in the chart.

*We thank the reviewer for this suggestion. We have reviewed all figure labels, legends, and captions for spelling errors and clarity. We have updated habitat site names to be capitalized, and all labels and captions are now clear.*

9.The paper points out that there is no significant difference in ASV richness between the skin and cloaca, and their taxa overlap, but the physiological microenvironments of the two are significantly different. The paper does not explain the ecological mechanism behind this taxonomic overlap. Could you tell me if there are any related hypotheses or plans for further validation?

*We appreciate this question from the reviewer. It is not known to what extent the cloaca and skin are significantly different microenvironments. We assume there are strong similarities between skin and cloaca, with both being oxygen limited environments with less sunlight exposure when basking then the carapace. There is also a clearer gradient/continuation allowing microbial transfer/movement from the external part of the cloaca to the skin, whereas there is more clear separation between the cloaca and the shell. To better clarify how body site differences exhibit continuous gradients versus hard categorical separation a more detailed study of the microbiomes across gradients could be examined, although this was not the focus of our study. We now include this suggestion for future work in the discussion. (Lines 253-260; 346-347).*

10.Inconsistent reference formatting: some references lack author names, publication years, or journal information, and some author name abbreviations are inconsistent. It is recommended to standardize the references according to the journal's format, fill in the missing information, and unify the formatting.

*We thank the reviewer for pointing out this inconsistency. All reference years have now been included. We have also better unified the formatting to follow ASM reference format, including a numbering scheme instead of alphabetical ordering.*

**Reviewer #2 (Comments for the Author):**

This manuscript systematically investigates the differences in microbial communities across various body sites of the Yellow Mud Turtle (*Kinosternon flavescens*), while also assessing the effects of habitat, age, and severity of shell disease. Using 16S rRNA sequencing, linear mixed-effects models, and PERMANOVA analyses, this study presents clear conclusions with significant ecological implications, particularly demonstrating innovation in linking variations among body sites with associations to Cyanobacteria. However, there are some flaws in experimental design and statistics, and some interpretations appear to extend beyond the direct support of the data. The detailed comments are as follows:

1. Lines 37-38: The abstract claims that bacterial community differences exist among habitat types. However, the results section (lines 146-147) indicates that no significant pairwise differences were detected among habitat sites after correcting for multiple testing. In the discussion (lines 247-262), the authors reference previous studies to support the effects of habitat. This may lead readers to overestimate the strength of evidence derived from this dataset. It is recommended that the conclusive statement in the abstract be revised to more accurately reflect the statistical evidence presented in the results.

*We thank the reviewer for pointing out this discrepancy. We agree the abstract has overstated habitat effects. While our PERMANOVA analysis detected overall habitat significance, no pairwise comparisons remained significant after correcting for multiple testing. We have therefore revised the relevant portion of the abstract as follows: "Using 16S rRNA amplicon sequencing of 64 turtle samples, we found bacterial community differences among body sites (cloaca, carapace, plastron, and skin), but weaker trends associated with habitat types (ephemeral or permanent-water ponds) and over age gradients (age 4–12+ years)." (Lines 39-41)*

2. Figure 3 (Heatmap): The clustering of samples in Figure 3 is overly dense, reducing readability. Additionally, although different phyla are color-coded, bacterial taxa from the same phylum are scattered throughout the rows, making intuitive interpretation of patterns at the phylum level difficult. To enhance the clarity and informational value of this figure, it is recommended to: (1) simplify or optimize the display of sample clustering, (2) group taxa from the same phylum adjacently, and (3) focus the main-text figures on key comparisons for improving readability.

*We thank the reviewer for their helpful comments on this figure. We have updated the heatmap to make it easier to interpret. Hierarchical clustering has been removed, and samples are now grouped by body site, with gaps added between the four body site groups to improve readability. We have retained row ordering by ASV from greatest to least abundance of our top 25 taxa to emphasize abundant ASV-level patterns. Phylum-level patterns are also included through corresponding row annotations. We have also updated the figure caption and legend to clarify the ordering of ASVs and to correctly reflect habitat site names, respectively. (Lines 223-229)*

3. Lines 249-250: As the authors indicated in the discussion, the relatively small sample size may have influenced some statistical outcomes. Nevertheless, the modest independent explanatory power (partial $R^2$) of the tested factors-most explaining less than 10% of the variation-suggests limited individual ecological effects. A brief discussion on the biological interpretation of these effect sizes, along with a simple visualization (e.g., variance partitioning), would enhance the clarity and ecological context of the β-diversity results.

*We appreciate the reviewer's suggestions. We agree that small sample size limits power, however the modest partial $R^2$ values referenced also reflect typical complexity in community ecology, indicating community level emergent effects from complex interactions. To make sure the biological context is interpreted accordingly, we have added additional text to clarify interpretations of the beta diversity results:*

*"Although individual factors explained some independent proportions of beta-diversity variation (partial $R^2 < 0.20$), these values represent typical ecological community complexity where multiple weaker factors are operating together resulting in larger emergent effects. While these factors exhibited statistical significance, most showed modest effect sizes (partial $R^2 < 0.10$), and body site explained the largest proportion (partial $R^2 = 0.16$ for Bray-Curtis beta-diversity) representing our most biologically meaningful result." (Lines 279-286)*

4. Lines 222-262: The discussion cites studies of other turtle species to support established patterns but does not sufficiently engage in a deeper comparison that incorporates the unique ecological traits of the study species (e.g., its strong terrestrial habits and arid habitat). It is recommended to utilize these comparisons to further elucidate the distinctive findings of this study. For example, could the patterns of body-site microbiome differentiation observing in *Kinosternon flavescens*, in comparison to the more aquatic species referenced, be linked to its unique life-history features such as terrestrial activity and exposure to dry conditions?

*We thank the reviewer for this important comment. We have added additional text into the Discussion highlighting differences between K. flavescens and other turtles and the difficulty in comparing our results with this species to all other taxa studied previously.* (Lines 296-299).

5. Lines 263-265: The text indicates that cyanobacterial abundance differs among groups categorized by shell pitting severity. However, this result only states that "a difference exists" without clarifying the specific pattern of change (e.g., whether abundance increases with worsening pitting). The discussion is too speculative regarding a functional association, such as photosynthetic commensalism, thus lacks clear trend data support.

*Thank you for this comment. ANCOM-BC2 analysis (carapace as reference) showed Cyanobacteria significantly more abundant on carapace than plastron (negative log fold change, $q < 0.05$; Supplemental Table 1), and also more detailed abundance data pairwise comparisons available in Supplementary File 4. We respectfully note that our ecological discussion linking carapace associated Cyanobacteria to sunlight exposure is grounded in Cyanobacteria's known photosynthetic capabilities and turtle anatomy, where the carapace would have more sunlight exposure, thereby representing a logical hypotheses and not unsupported speculation. We have made changes to the relevant results and discussion section to read as follows:*

*Results have been modified to include the following statement: "Cyanobacteria was seen to be significantly enriched in the carapace as compared to the plastron ($q < 0.05$)." (Lines 217-218)*

*We have also revised the Discussion as follows: "Cyanobacteria were significantly more abundant on the carapace than plastron." (Lines 312-313)*

6. Lines 335-339: The study includes age (7 groups) and shell pitting stage (5 groups) as factors. However, the number of individual turtles per specific age group or pitting stage is not provided. Given the total sample size of only 16 turtles, it is likely that some groups contain very few samples, which

can substantially reduce statistical power and affect the reliability of the results. It is suggested that this information be included (e.g., in a supplementary table), and the limitation noted in the discussion to assist readers in interpreting these results.

*Thank you for the suggestion. We have included an additional supplementary table giving the turtle sample size used for each factor examined. We have also included the following sentence in our Methods section on Sample Collection : "Turtle sample distribution across body site, habitat site, age group, and shell pitting status are summarized in Supplementary Table 2." We have also included language in the last paragraph of the discussion on sample size limitations and interpretation of results. (Lines 379-380; 341-349; Supplementary table 2 is included as a separate file).*

7. Lines 120-175, 214-221: Although the authors reported several statistically significant findings ($p < 0.05$), it would strengthen the discussion to differentiate more clearly between statistical and biological significance. Clarifying which effects are small but statistically detectable versus ecologically meaningful will improve the interpretive balance of the results.

*We thank the reviewer for the suggestion. We agree that it is important to differentiate between statistically significant and biologically meaningful results. We have made this context more clear with edits addressing comment 3, where small R2 values are part of a more complex community. We have also clarified that the differences in microbial communities between body sites is our strongest result (based on partial R2) and is likely to be the most ecologically meaningful with respect to the factors examined that shape K. flavescens microbiomes. These clarifications improve the interpretation of our results.*

*"While these factors exhibited statistical significance, most showed modest effect sizes (partial $R^2 < 0.10$), and body site explained the largest proportion (partial $R^2 = 0.16$ for Bray-Curtis beta-diversity) representing our most biologically meaningful result." (Lines 282-286)*

8. Lines 32-38, 98-99, 247-248, 340-343: Although age was included as a factor, sex was not analyzed due to small sample size. Given that sexual maturity can influence microbiome composition, especially in cloacal and skin regions, it would be valuable to briefly discuss how the absence of sex data might limit the interpretation of host-associated microbial differences.

*We thank the reviewer for these comments. We have clarified that the small sample size for the sex factor was due in part to younger turtles not having clear external sex characteristics for sex identification. To make this limitation clear we have also added additional language in our discussion related to this point:*

*"While understanding any potential sex differences may therefore be important due to physiological differences in sexually mature adults, sex was not included as a factor in our analyses because of the limited sample size of individuals with phenotypically identifiable sex. Many of the younger turtles sampled lacked clear external characteristics used for sex determination. Some of the unaccounted for community variation observed in our study may therefore be due to sex differences, which future studies with larger sample sizes could further evaluate." (Lines 270-276)*

9. Lines 496-498: The formatting of reference years is needed.

*Thank you for bringing this to our attention. The parentheses have been removed to standardize the year formatting.*

Re: Spectrum03664-25R1 (Microbiome Varies by Body Site in the Yellow Mud Turtle, *Kinosternon flavescens,* from the Chihuahuan Desert)

Dear Dr. Joseph D Madison:

Your manuscript has been accepted, and I am forwarding it to the ASM production staff for publication. Your paper will first be checked to make sure all elements meet the technical requirements. ASM staff will contact you if anything needs to be revised before copyediting and production can begin. Otherwise, you will be notified when your proofs are ready to be viewed.

**Data Availability:** ASM policy requires that data be available to the public upon online posting of the article, so please verify all links to sequence records, if present, and make sure that each number retrieves the full record of the data. If a new accession number is not linked or a link is broken, provide production staff with the correct URL for the record. If the accession numbers for new data are not publicly accessible before the expected online posting of the article, publication may be delayed; please contact ASM production staff immediately with the expected release date.

**Publication Fees:** For information on publication fees and which article types have charges, please visit our website. We have partnered with Copyright Clearance Center (CCC) to collect author charges. If fees apply to your paper, you will receive a message from no-reply@copyright.com with further instructions. For questions related to paying charges through RightsLink, please contact CCC at ASM_Support@copyright.com or toll free at +1-877-622-5543. CCC makes every attempt to respond to all emails within 24 hours.

**ASM Membership:** Corresponding authors may join or renew ASM membership to obtain discounts on publication fees. Need to upgrade your membership level? Please contact Customer Service at Service@asmusa.org.

**Embargo Policy:** A press release may be issued as soon as the manuscript is posted on the Spectrum Latest Articles webpage. The corresponding author will receive an email with the subject line "ASM Journals Author Services Notification" when the article is available online.

The ASM Journals program strives for constant improvement in our submission and publication process. Please tell us how we can improve your experience by taking this quick Author Survey.

Thank you for submitting your paper to Spectrum.

Sincerely,
Samara Mattiello
Editor
Microbiology Spectrum

Reviewer #1 (Comments for the Author):

Authors have addressed the comments. It can be accepted.

Reviewer #2 (Comments for the Author):

The author has revised the manuscript according to my review comments. I have no further issues.
