# Supplementary material for: Microbiome varies by body site in the yellow mud turtle, Kinosternon flavescens, from the Chihuahuan Desert
Source: Microbiol Spectr. 2026 Jul 20;14(9):e03664-25. doi: 10.1128/spectrum.03664-25 (PMC13532361; doi:10.1128/spectrum.03664-25)
Supplement: Supplementary file 2 [file spectrum.03664-25-s0010.docx]

**Legends for Supplementary Files, Supplementary Figures, and Supplementary Tables**

**File S1.** File containing metadata of all individual turtles captured (marked.turtle) and related swabs (SampleID) that were used in this study. Sampling location data and derived bacterial diversity metric values are also included.

**File S2.** Dada2 derived feature table with two sheets including ASVs by corresponding taxonomy (turtle_bodysite_microbiome_taxo) and also ASV counts by sample (turtle_bodysite_microbiome_feat).

**File S3.** FASTA file of all DNA sequences by corresponding ASVs.

**File S4.** File containing ANCOM-BC2 output with five sheets. One sheet includes the top five log-fold changed taxa (ancombc2_results_top5_all_pairw), and four additional sheets of the four separate pairwise comparisons by reference; carapace (ancombc2_results_carapace_refer), skin (ancombc2_results_skin_reference), plastron (ancombc2_results_plastron_refer), and cloaca (ancombc2_results_cloaca_referen).

**Figure S1.** Coverage given (pre-rarefaction) with sequence count plotted against richness. Each line represents a sample. Sample type by body site is given in the legend.

**Figure S2.** Relative abundance barplots showing the most commonly represented phyla, with individual sample IDs given on the x-axis. Barplots are arranged by body site (in heading for each panel). The phyla represented are listed in the legend with a corresponding color.

**Figure S3.** Principal coordinate analyses of bacterial community beta diversity using the Bray-Curtis dissimilarity metrics. Data groupings are given by (A) body site, (B) habitat site, (C) turtle shell pitting, and (D) turtle age. Data ellipses show a 95% confidence and assume a multivariate t-distribution. Color subgroups by factor are given in the corresponding legends.

**Table S1.** Turtle sampling distribution (N) across factors and categories.

**Table S2.** Top 5 ASVs by negative log-gold change (red) and top 5 ASVs by positive log-fold change (black), by pairwise comparison. All q-values given are multiple comparison corrected using Holm’s method. Significant q-values (q < 0.05) are given by an asterisk. In addition to ASV ID, phylum and genus (where applicable) are given. In the case no genus was identified, the lowest level of taxonomic identification is given in this column. Species is given in addition to genus when known. The respective body sites for the pairwise comparison are given at the top-left corner of each data subset.
